# A longitudinal assessment of the responsiveness of the ICECAP-A in a randomised controlled trial of a knee pain intervention

**DOI:** 10.1007/s11136-015-0980-0

**Published:** 2015-04-18

**Authors:** T. Keeley, H. Al-Janabi, E. Nicholls, N. E. Foster, S. Jowett, J. Coast

**Affiliations:** MRC Midland Hub for Trials Methodology Research, University of Birmingham, Edgbaston, Birmingham, B15 2TT UK; Health Economics Unit, School of Health and Population Sciences, Public Health Building, University of Birmingham, Edgbaston, Birmingham, B15 2TT UK; Arthritis Research UK Primary Care Centre, Research Institute for Primary Care and Health Sciences, Keele University, Keele, Staffordshire ST5 5BG UK

**Keywords:** Capability, ICECAP-A, Responsiveness, Psychometrics

## Abstract

**Purpose:**

The ICECAP-A is a simple measure of capability well-being for use with the adult population. The descriptive system is made up of five key attributes: Stability, Attachment, Autonomy, Achievement and Enjoyment. Studies have begun to assess the psychometric properties of the measure, including the construct and content validity and feasibility for use. This is the first study to use longitudinal data to assess the responsiveness of the measure.

**Methods:**

This responsiveness study was completed alongside a randomised controlled trial comparing three physiotherapy-led exercise interventions for older adults with knee pain attributable to osteoarthritis. Anchor-based methodologies were used to explore the relationship between change over time in ICECAP-A score (the target measure) and change over time in another measure (the anchor). Analyses were completed using the non-value-weighted and value-weighted ICECAP-A scores. The EQ-5D-3L was used as a comparator measure to contextualise change in the ICECAP-A. Effect sizes, standardised response means and *t* tests were used to quantify responsiveness.

**Results:**

Small changes in the ICECAP-A scores were seen in response to underlying changes in patients’ health-related quality of life, anxiety and depression. Non-weighted scores were slightly more responsive than value-weighted scores. ICECAP-A change was of comparable size to change in the EQ-5D-3L reference measure.

**Conclusion:**

This first analysis of the responsiveness using longitudinal data provides some positive evidence for the responsiveness of the ICECAP-A measure. There is a need for further research in those with low health and capability, and experiencing larger underlying changes in quality of life.

## Introduction

The ICEpop CAPability measure for Adults (ICECAP-A) is a new index measure of well-being with theoretical underpinnings in Sen’s [1–4] work on functioning and capability. Sen’s [2, 3] capability approach advocates an assessment of well-being that maintains a focus on what a person is able to do (capability), rather than what a person does (functioning). The capability approach encourages a broad evaluative space, which can include a person’s ability to achieve their basic requirements, such as living in good health, and more complex abilities, such as the ability to achieve things that are important to them, such as fulfilling social or professional roles [4]. Interest in the approach in health economics [5, 6], public health [7] and disability [8, 9] has increased in recent years.

The ICECAP-A was developed by Al-Janabi et al. [10] as a simple measure of capability well-being for use with the adult population. They conducted 36 semi-structured interviews using a purposively selected sample of the general population to identify what was important to people’s lives and well-being. The analysis identified five key attributes: Stability, Attachment, Autonomy, Achievement and Enjoyment. Poor health was found to be a key limiting factor in a person’s ability to attain these attributes. The second stage involved 18 semi-structured interviews to establish appropriate terminology for each of the five attributes identified. The final version of the ICECAP-A is shown in “Appendix 1”. The best–worst scaling method was used to estimate values for the measure [11].

Assessments of reliability [12], construct validity [13], content validity [14] and feasibility of use [15] of the measure have been completed with the general public and healthcare professionals. Responsiveness refers to the ability of an instrument to measure important or meaningful change [16] and is an important psychometric property of a measure [17]. Responsiveness is a key factor in deciding whether a measure will be appropriate for use in intervention studies.

The aim of this study is to assess the responsiveness of the ICECAP-A measure. This is the first study to assess the responsiveness of the ICECAP, using longitudinal outcome data from a randomised controlled trial.

## Methods

### Design, participants and setting

This responsiveness study was completed within the Benefits of Effective Exercise for knee Pain (BEEP) trial, a primary care, multi-centre, pragmatic randomised controlled trial whose aim was to compare improvement in pain and function outcomes from three physiotherapy-led exercise interventions for older adults with knee pain attributable to osteoarthritis [18]. Participants were recruited through either a general practice record search, a survey of older adults registered with participating practices, or screening patient referrals to participating physiotherapy services for knee pain. The inclusion/exclusion criteria aimed to select a population that was typical of those seen in primary care. Participants with serious pathology (inflammatory arthritis, malignancy, etc.), with previous hip or knee replacements on the affected side, on a surgical waiting list for knee replacement, in a nursing home, unable to attend clinic due to mobility issues or those in whom exercise was contraindicated, were excluded.

Participants were randomised to one of three interventions: usual physiotherapy care, individually tailored exercise and targeted exercise adherence, and patients were followed up at 3, 6, 9 and 18 months following randomisation [18]. The intervention arm that participants were randomised to was not used in this assessment of the responsiveness of the ICECAP-A.^1^ Rather, outcome measures administered to participants in the trial were used to form anchors against which the responsiveness of the ICECAP-A could be assessed. Data from baseline and the 6-month follow-up were used, since 6 months were the trial’s primary end point.

### Anchor selection

This analysis used anchor-based methods to explore the relationship between change over time in scores in the ICECAP-A and change over time in another measure (the anchor) [19]. The objective of an anchor-based analysis is to assess whether scores on the target measure change in the expected direction as indicated by changes in the scores on the anchor measure(s).

In line with recommendations by Revicki [19, 20], anchors were chosen based on: (a) the change correlation between the measures, (b) the cross-sectional correlations at baseline and follow-up between the measures, (c) whether the analysis using the anchor would increase the understanding of how the ICECAP-A measure responds to change in health and whether this would be of importance to investigators and researchers. It is recommended that multiple anchors are used [20].

An exploratory analysis of the correlations between the change scores of BEEP trial outcome measures was used to assist the choice of anchors for this analysis^2^ (“Appendix 2”). Based on this exploratory analysis, and on other points detailed above, the EuroQoL 5 Dimension Index (EQ-5D-3L), the Generalised Anxiety Disorder Assessment (GAD-7) and the Personal Health Questionnaire Depression Scale (PHQ-8) were chosen as anchors for this analysis. This was because the change scores of these anchors showed the strongest correlations with the ICECAP-A change scores and because baseline correlations between these anchors and the ICECAP-A were over 0.5. The use of these measures also has the benefit of including both physical and mental health domains. The Western Ontario and McMaster Universities Osteoarthritis Index (WOMAC) sub-scales showed very weak correlation in change scores, so were not used as anchors in this analysis.

### Anchor group formation

The EQ-5D-3L is a generic preference-based outcome measure, which measures health-related quality of life [21–24]. The descriptive system comprises of Mobility, Self-Care, Usual Activities, Pain and Discomfort, and Anxiety and Depression [25], with three response options in each dimension. It is scored via a preference-weighted algorithm, which for UK values produce a score between −0.59 and 1. The EQ-5D-3L has been extensively validated in numerous clinical settings [26–28]. The GAD-7 and the PHQ-8 are two short questionnaires that assess anxiety [29] and depression [30], respectively, which have validity portfolios [30, 31]. They are frequently used in research and have recognised values, above which anxiety or depression is indicated. The value range is from 0 to 21 for the GAD-7 and 0 to 24 for the PHQ-8, with a higher score indicating more severe anxiety and depression symptoms, respectively. Previous assessments of the construct validity of the ICECAP-A have shown that health-related quality of life and psychological or mental health status are related to ICECAP-A scores [32]. Therefore, some changes in ICECAP-A measures would be expected in response to changes in these measures.

Each anchor measure was used to divide the sample into: (1) those that had worsened between baseline and 6-month follow-up and (2) those that had improved between baseline and follow-up. Three anchors were used in this analysis. For the EQ-5D-3L, subgroups were formed using the minimally important change value of 0.074 [33]. Change groups were formed of participants who had changed by +or −0.074. Anchor groups from the GAD-7 and PHQ-8 score were not formed using a minimally important difference as no value could be found in the existing literature. A value of equal to or greater than ±2 was used to define the change groups for the GAD-7 and PHQ-8, which allowed adequate numbers in each of the change groups. For each anchor, a “no change” group was formed using the values between the improved and worsened groups. The numbers in each of the change groups, the mean change and change as a percentage of possible change is shown in Table 1.

**Table 1:** Numbers and mean change in anchor measures used

Anchor	Improved			Worsened		
	Number in group	Mean change (95 % CI)	Change as % of possible change	Number in group	Mean change (95 % CI)	Change as % of possible change
EQ-5D-3L	97	0.29 (0.25, 0.33)	18.2	38	−0.31 (−0.37, −0.24)	19.3
GAD-7	83	4.843 (4.04, 5.64)	23.1	43	−4.93 (−6.08, −3.78)	23.5
PHQ-8	92	4.473 (5.14, 3.81)	18.6	54	−4.254 (−3.46, −5.05)	17.7

### Value-weighted and non-weighted analyses

When assessing the responsiveness of a value-weighted measure, consideration needs to be given independently to both the descriptive system and the value weighting of the descriptive system [34]. The ability of the descriptive system to detect change in a construct is an essential precursor for the ability of a preference-based measure to accurately reflect preferences. If the analysis is solely completed using the preference-weighted scores, a conclusion might be made that the measure is not responsive; when in fact, the descriptive system of the measure is responsive, but the change is not valued [34].

For each anchor, two analyses are presented: (1) an analysis of the non-weighted descriptive system of the ICECAP-A and (2) an analysis of the value tariff scores. For the non-weighted sum score analysis and value tariff analysis, change was calculated in groups that improved and worsened. Non-weighted sum scores were calculated by summing ICECAP-A item scores, with four indicating full capability on an item and one indicating no capability on an item. The value tariff was calculated using the algorithm from Flynn et al. [35]. Change was measured both as actual change and change as a percentage of possible change, which was calculated by dividing the actual change by the range of the ICECAP-A measure and multiplying it by 100. The range of the measure for the non-weighted ICECAP-A score was 16 (5–20) and for the value tariff was 1 (0–1).

### Analyses

Two effect size (ES) statistics are reported for the change groups: a standard ES and the standardised response mean (SRM). The paired *t* test was used to test the null hypothesis that there has been no change in the mean response between baseline and follow-up.

For the GAD-7 and PHQ-8 analyses, the responsiveness of the EQ-5D-3L was additionally assessed as a comparator measure to add context to the ICECAP-A results. The use of the EQ-5D-3L as a comparator to the ICECAP-A was not designed to assess which measure performs “best”, as they are measures of two different constructs. Rather it was designed to increase the understanding of the size of changes in ICECAP-A scores in the context of another value (or preference)-based measure.

An assessment of the responsiveness of the individual ICECAP-A items was completed for each anchor measure using a response profile (the frequency of respondents answering each level for each item, at baseline and follow-up). Change in response profiles between baseline and follow-up was analysed for each level of each item to indicate which items were the “drivers” of change in the overall measure.

### A methodological note

The majority of responsiveness analyses of patient-reported outcome measures seek to assess how the scores of a measure change when the construct that the measure is designed to assess changes. This analysis was different and assessed the change in a capability measure when a change in health-related quality of life or mental health occurred. The ICECAP-A is the first, simple measure of capability well-being, potentially appropriate for use in trials testing health interventions. Other indicators of capability are not routinely used in health research. Therefore, quantifying change in capability, against which the responsiveness of the ICECAP-A measure could be assessed, was not possible. Other indicators of change must be used. From the data available for this research, it was possible to quantify change in both health-related quality of life and mental health.

While the use of health as an anchor was motivated by methodological considerations, when considering whether a capability measure is suitable for use in a health research setting researchers will likely want to know how it responds to changes in health, which in the vast majority of situations is the primary outcome. This analysis of change in a capability measure when a change in health occurs may be equally or more useful to health researchers. Health is one of many factors that affect a person’s capability scores on the ICECAP-A measure. Smaller change in capability scores would therefore be expected in response to changes in health.

## Results

### Participant characteristics

The characteristics of the BEEP trial participants used in this responsiveness analysis are presented in Table 2. A complete case analysis was completed, which included those who completed the ICECAP-A measure at both baseline and follow-up. The mean age of participants was 64, with a roughly equal proportion of male and female participants. The average ICECAP-A capability tariff values were higher (indicating higher capability) at both baseline and follow-up than values previously reported in the general population [36]. Participants reported mean EQ-5D-3L scores at baseline and follow-up, which were lower than the UK national average for this age group, indicating poorer health-related quality of life [37]. The GAD-7 and PHQ-8 scores did not indicate high levels of anxiety or depression within this sample [29, 30].

**Table 2:** BEEP trial sample characteristics, including mean scores and median scores

Attribute	Measure range	Sample size	Mean baseline values (SD)	Median value baseline (IQR)	Mean follow-up value (SD)	Median value follow-up (IQR)
Age mean		357	63.9 (9.83)			
Gender (% male)		357	49.3 %			
ICECAP-A tariff	0–1	355	0.89 (0.11)	0.91 (0.85, 0.97)	0.89 (0.12)	0.92 (0.85, 0.97)
EQ-5D-3L index	−0.59–1	351	0.64 (0.23)	0.69 (0.62, 0.76)	0.70 (0.22)	0.73 (0.69, 0.8)
GAD-7	0–21	344	3.14 (4.41)	1 (0, 4)	2.50 (3.91)	1 (0, 4)
PHQ-8	0–24	341	3.69 (4.44)	2 (1, 5)	2.99 (3.89)	2 (0, 4)

Table 2 shows that overall change in the ICECAP-A in this population, between baseline and 6-month follow-up, was negligible. This was also the case for other measures. Mean change can “hide” individual change, and when completing a responsiveness analysis, the range of change which is present in a sample is an important consideration. Analysis (see “Appendix 3”) showed that the majority of participants changed by less than 0.1 on the ICECAP-A measure. Therefore, this responsiveness analysis was completed in a population, which had high baseline capability and small changes between baseline and follow-up.

### Health-related quality of life: EQ-5D-3L anchor

Table 3 shows the change in non-weighted and value tariff ICECAP-A scores in groups that reported improved and worsened EQ-5D-3L scores. In groups that reported improved EQ-5D-3L scores ICECAP-A scores increased; in the groups that reported a worsening of EQ-5D-3L scores ICECAP-A scores decreased. The change in ICECAP-A scores was larger in the group that reported a worsening of EQ-5D-3L than an improvement. ES and SRM for those reporting an improvement in EQ-5D-3L were small for both the non-weighted and the value tariff scores; for those reporting a worsening in EQ-5D-3L scores, the ES and SRM were moderate or approaching moderate. Change as a percentage of possible change was smaller, in both the improved and the worsened groups, for the value tariff scores than for the non-weighted scores. The item-by-item analysis (“Appendix 4”) shows that in the group reporting improved EQ-5D-3L scores the largest changes were seen in Stability, Autonomy and Achievement, while in the group reporting a worsening the largest change was seen in Autonomy and Enjoyment.

**Table 3:** Mean change in non-weighted ICECAP-A scores and ICECAP-A tariff scores by EQ-5D-3L index anchor change groups (*n* = 341)

Anchor group	Baseline ICECAP-A	Follow-up ICECAP-A	Mean ICECAP-A change (95 % CI)	Change as a % of possible change	ES	SRM
Non-weighted ICECAP-A scores						
Improved	16.423	16.897	0.474** (0.123, 0.826)	3.2	0.20	0.27
No change	17.131	17.150	0.019 (−0.190, 0.229)	0.1	0.01	0.01
Worsened	16.895	15.842	−1.053** (−0.496, −1.609)	7.0	0.47	0.62
ICECAP-A tariff score						
Improved	0.863	0.884	0.021* (0.001, 0.041)	2.1	0.17	0.21
No change	0.898	0.895	−0.003 (−0.128, 0.007)	0.3	0.02	0.03
Worsened	0.890	0.836	−0.054** (−0.084, −0.024)	5.4	0.53	0.59

* Significant at the 5 % level, ** significant at the 1 % level

### Anxiety: GAD-7 anchor

Table 4 shows the change in non-value-weighted and value tariff ICECAP-A scores in groups that reported improved and worsened GAD-7 scores. In the group reporting an improvement in GAD-7 scores the ICECAP-A scores increased; in the group reporting a worsening of GAD-7 scores the scores decreased. The change in ICECAP-A scores is larger in the group reporting a worsening of their scores than in the group reporting an improvement of their scores. ES and SRM for those reporting an improvement in GAD-7 were small for both the non-weighted and the value tariff scores; for those reporting a worsening in GAD-7 scores, the ES and SRM were moderate or approaching moderate. Change as a percentage of possible change was smaller, in both the improved and the worsened groups, for the value tariff scores than for the non-weighted scores. The item-by-item analysis (“Appendix 5”) showed that in those reporting improved GAD-7 scores the largest changes were seen in Stability and Enjoyment, while for those reporting a worsening similar change were seen in all items apart from Autonomy.

**Table 4:** Mean change in non-weighted ICECAP-A scores and ICECAP-A tariff scores by GAD-7 anchor change groups (*n* = 335)

Anchor group	Baseline ICECAP-A	Follow-up ICECAP-A	Mean ICECAP-A change (95 % CI)	Change as a % of possible change	ES	SRM
Non-weighted ICECAP-A scores						
Improved	16.012	16.390	0.378 (−0.024, 0.780)	2.5	0.15	0.21
No change	17.430	17.569	0.139 (−0.05, 0.328)	0.9	0.07	0.10
Worsened	16.442	15.279	−1.163** (−1.789, −0.537)	7.7	0.53	0.57
ICECAP-A tariff scores						
Improved	0.844	0.864	0.020 (0.002 0.042)	2.0	0.14	0.20
No change	0.913	0.917	0.004 (−0.003, −0.011)	0.4	0.04	0.07
Worsened	0.863	0.792	−0.071** (−0.11, −0.032)	7.1	0.58	0.55

* Significant at the 5 % level, ** significant at the 1 % level

The use of the EQ-5D-3L as a comparison shows differences from the ICECAP-A analysis (Table 5). The size of the change as a percentage of possible change and the SRM and ES were similar for both the weighted and the non-weighted analyses; however, the directional pattern of change was different. Change in the ICECAP-A was greater for those reporting a worsening of anxiety (7.7 vs 2.5 % in the non-weighted scores). The reverse of this was found in the EQ-5D-3L analysis (8.5 vs 2.2 % in the non-weighted scores).

**Table 5:** Mean change in non-weighted EQ-5D-3L scores and EQ-5D-3L index scores by GAD-7 anchor change groups (*n* = 335) for comparison

Anchor group	Baseline EQ-5D-3L scores	Follow-up EQ-5D-3L scores	Mean EQ-5D-3L change (95 % CI)	Change as a % of possible change	ES	SRM
Non-weighted EQ-5D-3L scores						
Improved	8.16	7.308	−0.852** (−1.142, −0.561)	8.5	0.56	0.65
No change	7.421	7.015	−0.406** (−0.56, −0.253)	4.1	0.35	0.37
Worsened	7.878	8.097	0.219 (−0.69, 0.251)	2.2	0.14	0.14
EQ-5D-3L index scores						
Improved	0.585	0.686	0.101** (0.052, 0.149)	6.3	0.39	0.46
No change	0.667	0.717	0.05** (0.022, 0.078)	3.1	0.24	0.24
Worsened	0.614	0.616	0.002 (−0.079, 0.083)	0.1	0.01	0.01

* Significant at the 5 % level, ** significant at the 1 % level

### Depression: PHQ-8 anchor

In the group reporting an improvement in PHQ-8 scores ICECAP-A scores increased, while in the group reporting a worsening of PHQ-8 scores ICECAP-A scores decreased (Table 6). The magnitude of change and the ES and SRM were larger in the group who reported a worsening of anchor scores than in the group reporting an improvement. Change as a percentage of possible change was smaller for the value tariff than the non-weighted score. The item-by-item analysis (“Appendix 6”) showed that in those reporting a worsening on PHQ-8 scores the largest change was seen in Enjoyment, while in those reporting an improvement the largest change was found in Stability and Achievement.

**Table 6:** Mean change in non-weighted ICECAP-A scores and ICECAP-A tariff scores by PHQ-8 anchor change groups (*n* = 331)

Anchor group	Baseline ICECAP-A	Follow-up ICECAP-A	Mean ICECAP-A change (95 % CI)	Change as % of possible change	ES	SRM
Non-weighted ICECAP-A scores						
Improved	16.217	16.576	0.359 (−0.003, 0.720)	2.3	0.15	0.2
No change	17.486	17.616	0.13 (−0.077, 0.336)	0.8	0.07	0.09
Worsened	16.629	15.759	−0.87** (−1.398, −0.343)	5.8	0.37	0.45
ICECAP-A tariff scores						
Improved	0.852	0.866	0.014 (−0.005, 0.032)	1.4	0.11	0.15
No change	0.917	0.92	0.003 (−0.006, 0.011)	0.3	0.02	0.03
Worsened	0.872	0.825	−0.048** (−0.078,−0.017)	4.8	0.39	0.43

* Significant at the 5 % level, ** significant at the 1 % level

The use of the EQ-5D-3L as a reference measure shows differences from the ICECAP-A analysis (Table 7). The size of the change as a percentage of possible change and the SRM and ES were larger for the EQ-5D-3L analysis in comparison with the ICECAP-A analysis. The directional pattern of change was different. Change in the ICECAP-A was greater for those reporting a worsening of anxiety than for those reporting an improvement (5.8 vs 2.3 % in non-weighted scores). The reverse of this was found in the EQ-5D-3L analysis (2.2 vs 8.5 % in non-weighted scores).

**Table 7:** Mean change in EQ-5D-3L scores by PHQ-8 anchor change groups (*n* = 326) for comparison

Anchor group	Baseline EQ-5D-3L	Follow-up EQ-5D-3L	Mean EQ-5D-3L change (95 % CI)	Change as % of possible change	ES	SRM
Non-weighted EQ-5D-3L scores						
Improved	8.161	7.309	−0.852** (−1.142,−0.561)	8.5	0.56	0.64
No change	7.421	7.015	−0.407** (−0.56,−0.253)	4.1	0.35	0.36
Worsened	7.878	8.097	0.219 (−0.251, 0.69)	2.2	0.14	0.15
EQ-5D-3L index scores						
Improved	0.559	0.659	0.1** (0.05, 0.149)	6.3	0.37	0.42
No change	0.689	0.744	0.056** (0.029, 0.082)	3	0.33	0.31
Worsened	0.653	0.621	−0.031 (−0.098, 0.036)	2	0.13	0.13

* Significant at the 5 % level, ** significant at the 1 % level

## Discussion

This is the first analysis to assess the responsiveness of the ICECAP-A measure using longitudinal data from a randomised controlled trial. The results provide some positive evidence for the responsiveness of the ICECAP-A measure. Small changes in the ICECAP-A scores were seen in response to changes in health-related quality of life and mental health status. In the GAD-7 (anxiety) and PHQ-8 (depression) analyses, ICECAP-A change was of a comparable size to change in the EQ-5D-3L reference measure, but the pattern of change showed differences. Differences were found between the value-weighted and non-value-weighted ICECAP-A scores, with non-weighted scores being slightly more responsive.

### Discussion of principal findings

A number of important findings should be highlighted: (1) a non-perfect relationship exists between health and capability scores, (2) differences exist between the non-value-weighted and value-weighted ICECAP-A scores and (3) the magnitude of change was similar for the ICECAP-A and the EQ-5D-3L reference measure, with differences in the patterns of change.

Results indicate a non-perfect relationship between change in health and change in capability. In this context, correlation between anchor change scores and ICECAP-A change scores was weak, and change in ICECAP value tariff was small in comparison with the change in the anchors. A change in health represents a change in one of a number of factors that affects a person’s capability [10, 38]. Therefore, the impact that small changes in health have upon change in the descriptive system of the ICECAP-A measure may be small. The results presented above provide supporting evidence for this conclusion.

A comparison between the non-value-weighted ICECAP-A scores and the value-weighted ICECAP-A tariff change scores shows that change as a percentage of possible change is smaller for the value-weighted tariff scores than for the non-value-weighted scores. This indicates that when the value tariff was applied to the non-weighted scores the magnitude of change was reduced. The value tariff for the ICECAP-A was calculated using best–worst scaling [10]. There are differences in the value attached to change between the different within item levels. Significant value is attached to change between the levels “none” and “a little”, while little value is attached to changes between “a lot” and “all”.

This responsiveness analysis was completed in a “high capability” population (mean baseline ICECAP-A tariff score was 0.89). The item-by-item analysis shows that the majority of change in this population occurred among respondents whose answers switched between the top two levels of both measures. Therefore, the majority of change occurred at the top of the measure. When applying the value tariff, these changes are of less value and contribute less weight to the overall tariff score than changes at the bottom of the measure. Consequently, these changes at the top of the measure held less weight in the value-weighted tariff score than they did in the non-value-weighted score. These results show that the responsiveness of the descriptive system is reduced when the tariff is applied because changes at the “top end” are not strongly valued.

The use of the EQ-5D-3L as a reference measure showed that the size of change, the ESs and the SRMs were similar for the non-weighted ICECAP-A scores as they were for the non-weighted EQ-5D-3L scores. While the size of change and the signal to noise ratios were similar, the pattern of change was different. The ICECAP-A showed greater change in groups whose mental health had deteriorated, than improved. The opposite was found for EQ-5D-3L. This may have been due to the high ICECAP-A score found at baseline, leaving less scope for the scores on the measure to change in response to an improvement in health.

### Strengths and weaknesses of the research

There are a number of strengths of this research. The data provided from the trial were well-completed, and the availability of data from baseline and 6-month follow-up allows longitudinal analysis of the ICECAP-A measure. The anchor-based methodology used to assess the responsiveness of the measure represents best practice in the field.

There are some limitations that are worth noting. The use of a trial population with high baseline capability and showing small changes during the 6-month follow-up in capability and health domains measured by anchors selected results in some limitations in this analysis. The predominance of health measures available for use as anchors, which would likely be the case in most effectiveness trials of health interventions, reduces the scope of the analysis by not allowing assessment of how the measure responds to changes in other determinants of capability. The absence of minimally important change values for the GAD-7 and PHQ-8 meant that values used to define the groups were chosen based on securing adequate numbers in each of the change groups. The use of different values may have led to different results.

### Implications of the work

The evidence of responsiveness presented in this paper adds to the psychometric portfolio of the ICECAP-A measure. These results should allow researchers to use the ICECAP-A measure with greater confidence than has previously been the case. Responsiveness is a context-specific property, and therefore, caution should be exercised in generalising these results.

The ICECAP measures have been highlighted by the National Institute for Health and Care Excellence and the Social Care Institute for Excellence as broad preference-based measures, which are potentially suitable for use in social care research [39]. This evidence provides an initial indication that the ICECAP-A measure may be responsive in healthcare research. This highlights the need for responsiveness evidence in other research areas, such as social care.

### Directions for future research

Future research into the responsiveness of the ICECAP-A measure would benefit from anchor measures assessing a greater variation of constructs. The use of measures which provide information on connectedness, happiness, independence or hope, which are rarer in trials testing healthcare interventions, would add to our understanding of the responsiveness of the ICECAP-A measure. A further area for future research would be within populations which have lower baseline capability than seen in this study and populations who experience larger changes in capability over time.

## Conclusion

This paper provides the first evidence of responsiveness of the ICECAP-A measure. Small changes in ICECAP-A scores were seen in response to changes in physical and psychological health. These results will be of interest to both those looking to use the measure in research and those currently assessing the psychometric properties of the measure.

## Appendix 2

An exploratory analysis of correlations between change scores of the outcome measures included in the BEEP trial.

**Table:**

	ICECAP-A score	EQ-5D-3L index	WOMAC pain	WOMAC stiffness	WOMAC function	GAD-7	PHQ-8
ICECAP-A tariff	1.00						
EQ-5D-3L score	**0.255**	1.00					
WOMAC pain	−0.055	−0.402	1.00				
WOMAC stiffness	−0.151	−0.236	0.507	1.00			
WOMAC function	−0.103	−0.380	0.737	0.592	1.00		
GAD-7	−**0.205**	−0.202	0.109	0.040	0.129	1.00	
PHQ-8	−**0.190**	−0.232	0.057	0.092	0.090	0.511	1.00

Highlighted in bold are the measures that were chosen as anchors for use in this analysis

## Appendix 3

Frequency distribution of change in ICECAP-A tariff score.

## Appendix 4

The item-by-item analysis showed that in the group of participants reporting a worsening of EQ-5D-3L index scores there was a substantial reduction, of 21 points, in the percentage of participants reporting full capability on the Autonomy and Enjoyment items. In the group reporting an improvement in EQ-5D-3L the largest increases were seen in Stability, Autonomy and Achievement.

**Table:** ICECAP-A response profile for worsened EQ-5D-3L index scores

	Baseline profile					6-month follow-up profile					Change between baseline and follow-up				
	Stab	Attach	Auto	Achieve	Enjoy	Stab	Attach	Auto	Achieve	Enjoy	Stab	Attach	Auto	Achieve	Enjoy
Level 1	0	0	0	0	0	3	0	0	0	0	+3	0	0	0	0
Level 2	10	5	3	13	8	18	13	5	18	18	+8	+8	+2	+5	+10
Level 3	58	29	37	63	45	58	29	55	63	55	0	0	+18	0	+10
Level 4	32	66	60	24	47	21	58	39	18	26	−11	−8	−21	−6	−21

**Table:** ICECAP-A response profiles for improved EQ-5D-3L index scores

	Baseline profile					6 month follow-up profile					Change between baseline and follow-up				
	Stab	Attach	Auto	Achieve	Enjoy	Stab	Attach	Auto	Achieve	Enjoy	Stab	Attach	Auto	Achieve	Enjoy
Level 1	4	0	0	0	0	1	1	1	1	1	−1	+1	+1	+1	+1
Level 2	15	8	3	13	10	9	5	4	10	9	−6	−3	+1	+3	−1
Level 3	55	33	43	62	52	56	31	28	54	50	+1	−2	−15	−8	−2
Level 4	26	59	54	25	38	34	63	67	35	39	+8	+4	+13	+10	+1

## Appendix 5

The item-by-item analysis showed that in the group of respondents reporting a worsening of GAD-7 scores there was a change of between 9 and 14 points in the percentage of respondents answering full capability for all items expect autonomy, which showed little change. In the group reporting an improvement in GAD-7 scores increase of 9 and 6 points were seen in the percentage of participants answering full capability for Stability and Enjoyment items.

**Table:** ICECAP-A response profile for worsened GAD-7 health status

	Baseline profile					6-month follow-up profile					Change between baseline and follow-up				
	Stab	Attach	Auto	Achieve	Enjoy	Stab	Attach	Auto	Achieve	Enjoy	Stab	Attach	Auto	Achieve	Enjoy
Level 1	5	0	0	0	0	7	0	2	5	5	+2	0	+2	+5	+5
Level 2	11	9	7	12	12	30	21	7	16	16	+19	+12	0	+4	+4
Level 3	58	30	33	65	53	49	33	30	65	58	−9	+3	−3	0	+5
Level 4	26	60	60	23	35	14	46	60	14	21	−12	−14	0	−9	−14

**Table:** ICECAP-A response profile for improved GAD-7 health status

	Baseline profile					6-month follow-up profile					Change between baseline and follow-up				
	Stab	Attach	Auto	Achieve	Enjoy	Stab	Attach	Auto	Achieve	Enjoy	Stab	Attach	Auto	Achieve	Enjoy
Level 1	4	0	0	0	0	0	1	0	0	1	−4	+1	0	0	+1
Level 2	19	11	5	17	16	18	8	2	12	12	−1	−3	−3	−5	−4
Level 3	59	32	38	65	60	55	32	36	69	57	−4	0	−2	+4	−3
Level 4	18	57	57	18	24	27	58	61	19	30	+9	+1	+4	+1	+6

## Appendix 6

The item-by-item analysis shows that in the group reporting a worsening of PHQ-8 health scores a substantial of 23 points is seen in the percentage of people reporting full capability on the Enjoyment item. In the group reporting an improvement in PHQ-8 health status increases of 7 and 14 percentage points were seen in the proportion of participants reporting full capability on the Stability and Achievement items, respectively.

**Table:** ICECAP-A response profile for worsened PHQ-8 health status

	Baseline profile					6-month follow-up profile					Change between baseline and follow-up				
	Stab	Attach	Auto	Achieve	Enjoy	Stab	Attach	Auto	Achieve	Enjoy	Stab	Attach	Auto	Achieve	Enjoy
Level 1	2	0	0	0	0	4	2	0	2	4	+2	+2	0	+2	+4
Level 2	15	6	4	15	13	22	14	4	13	16	+7	+8	0	−2	+3
Level 3	53	35	35	59	44	56	33	33	67	60	+3	−2	−2	+8	+16
Level 4	30	59	61	26	43	18	51	64	18	20	−12	−8	+3	−8	−23

**Table:** ICECAP-A response profile for improved PHQ-8 health status

	Baseline profile					6-month follow-up profile					Change between baseline and follow-up				
	Stab	Attach	Auto	Achieve	Enjoy	Stab	Attach	Auto	Achieve	Enjoy	Stab	Attach	Auto	Achieve	Enjoy
Level 1	2	0	0	0	0	2	0	0	1	1	0	0	0	+1	+1
Level 2	20	12	6	15	14	15	10	5	13	12	−5	−2	−1	−2	−2
Level 3	51	28	39	70	53	48	31	37	57	48	−3	+3	+2	+13	−5
Level 4	27	60	55	15	33	34	59	58	29	29	+7	−1	+3	+14	−4

## Footnotes

1 Therefore, this paper makes no statement about the effectiveness of the intervention within this trial.

2 Please note that not all measures included in the BEEP trial were used in the analysis of ICECAP-A responsiveness reported in this paper. This exploratory analysis identified measures to be used as anchors, based on predefined criteria recommended by Revicki.
